# Bringing patient-centered tuberculosis diagnosis into the light of day

**DOI:** 10.1186/s12916-017-0992-4

**Published:** 2017-12-20

**Authors:** J. Lucian Davis

**Affiliations:** 1 Department of Epidemiology of Microbial Diseases, Yale School of Public Health, 60 College Street, Room 620, New Haven, Connecticut 06520-8034 USA; 2 Pulmonary, Critical Care, and Sleep Medicine Section, Yale School of Medicine, New Haven, Connecticut USA

**Keywords:** Tuberculosis, Diagnosis, Prognosis, Fluorescence microscopy, Patient-centered care

## Abstract

In 2015, the WHO End TB Strategy laid out ambitious goals to dramatically reduce tuberculosis (TB) deaths, incidence, and catastrophic costs through research, bold new strategies, and patient-centered care. In this commentary, recent evidence on sputum collection strategies for smear microscopy is reviewed, and the argument is made that redesigning smear microscopy as a patient-centered service offers the only realistic and widely available strategy to advance TB diagnostic care towards the initial End TB Strategy goals laid out for 2025. Finally, the successful adoption of same-day sputum smear microscopy as a model for patient-centered TB care is suggested to be synergistic with and to form part of the scale-up of new TB diagnostic tools.

Please see related article: https://bmcmedicine.biomedcentral.com/articles/10.1186/s12916-017-0947-9

## Background

The last decade has produced major advances in diagnostic testing for active tuberculosis (TB), including the approval of several novel diagnostic assays by the World Health Organization (WHO) following rigorous evidence-based reviews [1, 2]. Unfortunately, these technologies remain inaccessible to the vast majority of patients undergoing TB evaluation in most high-burden countries because of their high cost and other implementation challenges. Thus, the de facto standard of care remains sputum smear microscopy, a 19th-century technique that involves smearing expectorated sputum on a glass slide, staining it, and examining it for acid-fast bacilli using light or fluorescence microscopy. While it is widely recognized that smear microscopy is less sensitive and more labor intensive than newer diagnostic tools, a much more important limitation of microscopy is that it is usually implemented in ways that ignore the needs and preferences of patients. For example, in most settings, sputum smear microscopy still involves serial collection of two or more sputa over 2 or more days, with at least one sample obtained by overnight or early morning collection. Unfortunately, diagnostic strategies that require multiple clinic visits have been repeatedly associated with high rates of diagnostic drop-out and catastrophic costs (i.e., exceeding 20% of annual household income) for patients undergoing evaluation for TB [3–5]. While WHO has issued several policies designed to streamline smear microscopy to allow collection of two sputum specimens on the same day [6, 7], this approach has not been widely adopted. Thus, a bold new TB diagnostic strategy must now be considered if the global community is to dramatically reduce TB deaths, incidence, and catastrophic costs by 2025 as called for by the WHO End TB Strategy. To achieve this, the most realistic and immediately scalable diagnostic policy is to make sputum smear microscopy more efficient and patient centered [8, 9].

### Recent evidence

Two recent articles provide welcome data to advance this agenda. In an article published in *BMC Medicine* on October 27, 2017, Murphy et al. [10] reported the results of a secondary analysis of clinical trial data to answer the question, “Are early-morning sputum samples superior to spot samples for diagnosis of active TB and monitoring for adverse outcomes of TB treatment?” The 1931 study participants were all enrolled in REMoxTB, a Phase III, randomized controlled trial that compared two moxifloxacin-based regimens to shorten the treatment of active, drug-susceptible TB against the standard 6-month regimen. The authors examined the results of smear microscopy, solid culture, and liquid culture performed on 1115 paired spot and early morning sputa collected at baseline for TB diagnosis, and on 2995 paired spot and early morning sputa collected at follow-up for TB treatment monitoring. They found that early morning samples did not significantly improve the performance of spot samples for TB diagnosis or prediction of unfavorable outcomes of TB treatment (i.e., failure and relapse).

In another article published in the August 2017 issue of *Lancet Global Health*, Datta et al. [11] described the findings of a systematic review and meta-analysis of sputum collection methods for TB diagnosis. The authors identified 23 eligible studies, including 8967 participants, and examined 19,252 sputum samples to determine which non-invasive sputum collection methods are most effective at increasing the diagnostic yield for active TB. Complementing the findings of the REMoxTB secondary analysis, this systematic review showed that neither early morning nor overnight sputum collection increased the yield of microscopy or culture compared with spot sputum collection. Although 24-h pooled sputum collection was associated with increased detection of TB, brief instruction of participants on how to produce a good quality sputum specimen just prior to expectoration provided similar results without requiring a second clinic visit. Furthermore, sputum instruction was associated with increases in yield of a magnitude similar to those produced by molecular testing with the Xpert MTB/RIF assay.

The above studies, incorporating data from a wide range of low- and middle-income countries, supplement substantial prior evidence showing that collecting an early morning specimen for acid-fast bacilli smear microscopy after an initial spot specimen adds very little diagnostic yield beyond that provided by collecting a second spot specimen on the day of presentation [7]. Nevertheless, promoting adoption of such patient-centered approaches to sputum smear microscopy is challenging given the large number of competing TB diagnostic priorities, including the introduction of new diagnostic tools and case-finding strategies. However, there are at least three important points from these and other recent studies that could foster wider dissemination of patient-centered diagnostic strategies incorporating sputum smear microscopy and Xpert MTB/RIF. Firstly, by shortening TB evaluation to one visit, TB programs may be able to achieve an up to 60% reduction in the diagnostic drop-out rate [12]. Secondly, same-day diagnostic strategies have great potential to reduce the catastrophic costs of TB care, not only for those undergoing evaluation and who are ultimately diagnosed with TB, but also for the much greater number who are found not to be infected. Thirdly, the elimination of extra clinic visits following early-morning sputum collection may free time for clinic staff to focus on three higher yield strategies, namely (1) instructing every patient on how to produce a good-quality sputum sample; (2) examining specimens and providing results on the same day; and (3) initiating treatment for those testing positive on the same day as diagnosis. Mathematical models suggest that scale-up of such patient-centered ‘test and treat’ strategies for smear microscopy could reduce TB incidence as effectively as molecular testing with the Xpert MTB/RIF assay, and that combined scale-up of same-day microscopy and Xpert MTB/RIF could decrease TB mortality by 44% over a decade [13, 14].

## Conclusions

Taken together, these data highlight same-day smear microscopy as an immediately scalable approach to diagnostic evaluation for TB that links patient-centeredness and quality of care through enhanced efficiency and reduced cost [15]. Both as a short-term TB diagnostic strategy and as a long-term model for delivery of patient-centered TB care, same-day microscopy is an approach well worth bringing out of the shadows of early morning sputum collection and into the light of day.
